# Humanitarian funding contraction and the risk of cost-related forgone care: a proposed financial-protection transition framework for internally displaced communities in Mogadishu, Somalia

**DOI:** 10.1080/16549716.2026.2755139

**Published:** 2026-10-29

**Authors:** Ahmed Omar Siyad, Abdukadir Mohamed Hassan

**Affiliations:** Department of Intensive Care Medicine, Dr. Sumait Hospital, SIMAD University, Mogadishu, Somalia

**Keywords:** Universal health coverage, unmet healthcare need, strategic purchasing, service continuity, adaptive social protection

## Abstract

Somalia’s humanitarian health response is contracting within a fragmented, aid-dependent system in which displaced households face direct payments and substantial access costs. National and humanitarian reports document major funding gaps and service reductions, but we identified no published study that directly estimates whether the recent funding contraction has increased household spending, delays in care, treatment interruption or cost-related forgone care among internally displaced communities in Mogadishu. This Current Debate is a purposive policy analysis based on selected peer-reviewed studies, Somali policy documents, humanitarian reports and international guidance; it is not an empirical study or a systematic review. We distinguish financial hardship among households that obtain care from cost-related financial exclusion, a broader construct encompassing delayed, incomplete or completely forgone needed care due to unaffordable direct or access costs. We propose a testable transition framework for both planned service reductions and abrupt withdrawal, linking service-gap assessment and essential-service prioritisation to funded protection from charges, time-limited bridge arrangements, appropriately governed purchasing, medicine and transport support, adaptive social protection and community accountability. A minimum viable monitoring set should combine indicators of service continuity, prescription fill, referral completion and unauthorised charges with household-level distress financing and should report cost-related delayed, incomplete and completely forgone care separately. The proposed pathways and interventions are evidence-informed hypotheses and policy options requiring local co-design, costing, feasibility assessment and prospective evaluation; they should not be interpreted as evidence that humanitarian funding contraction has already caused cost-related forgone care in Mogadishu.

## Paper Context

- **Main findings**: National evidence documents Somalia’s humanitarian health-service contraction, but no direct evidence establishes its effect on forgone care among displaced Mogadishu households.
- **Added knowledge**: This Current Debate proposes a testable framework linking planned and abrupt funding withdrawal to separate hardship and delayed, incomplete or completely forgone care indicators.
- **Global health impact for policy and action**: Funding reductions demand financed service continuity, explicit clinical priorities, accountable purchasing and monitoring that detects both hardship and exclusion.

## Background

The 2026 World Health Organization (WHO) Health Emergency Appeal reports that external financing supports 95% of Somalia’s health budget, with 618 facilities (51 district hospitals, 413 health centres and 154 primary health units) at risk of closure [1]. In September 2025, the Somalia Health Cluster received US$26.7 million (22%) of US$122.4 million required and reported hospital, primary-care and mobile-team service reductions [2]. These operational reports indicate national continuity threats, including Benadir, but not household-level effects in Mogadishu.

Displaced households in Mogadishu face transport burdens and informal settlement gatekeeping [3,4]. Mogadishu–Baidoa evidence reports transport difficulties, waiting times and medicine shortages but is not Mogadishu-specific [5]. No identified study estimates whether the 2025–2026 contraction increased spending, care delays, treatment interruption or cost-related forgone care among Mogadishu’s displaced households. Expenditure measures miss people priced out of care [6–9].

This Current Debate proposes a testable transition framework integrating service-contraction management, dual measurement of financial hardship and exclusion, and governance safeguards; it does not estimate causal effects [3,6,7]. Its contribution is synthesis rather than a new financing instrument.

## Approach

We conducted purposive policy analysis, not systematic synthesis. Searches updated to 30 August 2026 covered PubMed, Google Scholar and WHO, Somali health-authority, Health Cluster, statistics-bureau, World Bank and Overseas Development Institute repositories. We searched English-language material from January 2015–August 2026, retaining older relevant guidance. Terms covered funding cuts/service contraction, financial protection, out-of-pocket/catastrophic/impoverishing expenditure, forgone/delayed care, unmet need, displacement, Mogadishu/Somalia, purchasing, user fees, social protection, governance and data protection.

Peer-reviewed studies covered financial access, displacement and health financing; Somali policy/humanitarian documents covered financing, service reductions and operational constraints; guidance informed measurement and policy mechanisms. Original sources were prioritised for numerical claims. No directly comparable causal estimates were identified. For example, national service-reduction reports showed continuity threats, whereas Mogadishu–Baidoa access findings were treated as contextual, not evidence of Mogadishu household effects. Where sources differed in scope, period or methodological strength, claims followed the most directly applicable evidence without inferring causality. No systematic screening, risk-of-bias assessment, evidence grading or meta-analysis was undertaken; proposed actions remain recommendations requiring local testing.

## The measurement blind spot: distinguishing hardship, exclusion and related constructs

Financial hardship occurs when needed care is obtained but direct spending causes catastrophic expenditure, impoverishment or distress financing, such as borrowing, selling an asset or drawing on essential savings [6,7]. These measures can miss people who forgo care because they cannot pay. WHO guidance covers need, care receipt, delayed/incomplete receipt and reasons for non-use [8]. Here, a cost-related access barrier is any affordability-related obstacle affecting a reported care-need episode; financial exclusion is the broader construct, classified as delayed care, incomplete care or completely forgone care. Treatment interruption denotes disruption of required ongoing care. In an analysis of the 2022 Somalia Integrated Household Budget Survey, approximately 32% of individuals reporting a healthcare need did not use medical care, while 48.5% of those who did not receive care cited affordability as the main reason. These findings predate the 2025 humanitarian funding contraction and indicate pre-existing access barriers rather than the effects of funding cuts [9,10].

The classification unit for monitoring is the care-need episode. A single episode may meet both constructs: someone may pay for a consultation using distress financing yet be unable to afford a prescribed test or medicines, producing both financial hardship and incomplete financial exclusion; the unaffordable component should be recorded. Episode outcomes should remain distinct. Person-level indicators should report people with at least one affected episode among those reporting at least one need, while household-level indicators should report households with at least one affected member among households reporting a need; episode counts should remain available so recurrent episodes are not obscured. Cost mechanisms should be disaggregated rather than collapsed into ‘affordability’ [6–10].

The policy options draw on fragile-setting financing, humanitarian allocation, adaptive social protection and user-fee approaches [11–14]; their feasibility in Mogadishu remains untested.

## Hypothesised pathways and alternative explanations

Four hypothesised pathways link contraction to households: fewer facilities or outreach increase transport costs and referral failure [1–5]; commodity or diagnostic gaps shift purchases to private providers and increase household costs [3,5]; unfunded operations create charges and upfront payments delay emergency care [15]; and reduced community-health-worker/outreach capacity disrupts information, continuity and referrals [16]. Pathways may interact.

Attribution is complicated by drought, food-price and income pressures, conflict and insecurity, new displacement, outbreaks and provider changes [1,2]. Prospective evaluation should use pre-reduction baselines where feasible, record concurrent shocks and compare differently exposed catchments, rather than attributing changes to funding contraction. Exposure should be prespecified using observable measures such as percentage reduction in financing, lost service days, reduced commodity-support and mobile/outreach closure; components should be reported separately and any composite transparently weighted. Figure 1 summarises this hypothesised pathway.

**Figure 1.**
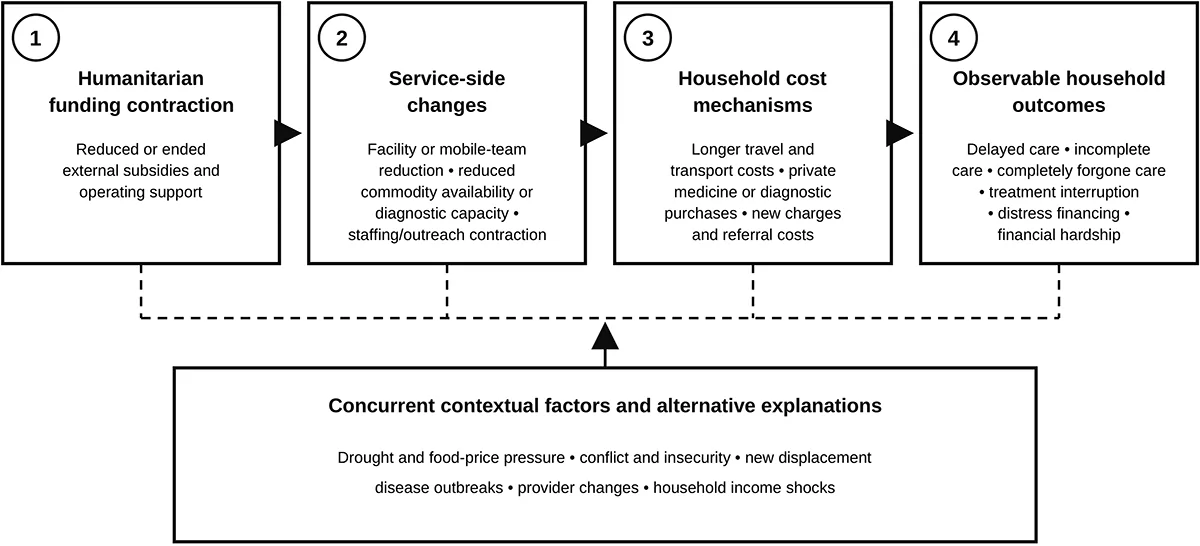
Proposed pathway from humanitarian funding contraction to household financial-access outcomes. Solid arrows indicate hypotheses, not established causal effects; contextual factors may independently affect services, costs and care-seeking.

## A financial-protection transition agenda under fiscal constraint

For planned reductions, authorities, funders, partners, providers and displaced-community representatives should map the affected package, catchment, staffing, commodities, referrals, subsidies and replacement costs before support ends. Abrupt withdrawal requires a rapid-response pathway: map lost services and affected populations, prioritise time-sensitive gaps, restore critical functions temporarily where feasible, and retrospectively identify displaced groups whose access was disrupted. The Essential Package of Health Services and Somalia’s investment framework can guide prioritisation [3,17]. When resources cannot cover all services, protection priorities should be explicit and transparent within available financing, considering preventable mortality/severe morbidity, clinical urgency, continuity of chronic treatment, maternal/newborn/child health, outbreak control, equity including disability/displacement, and the consequences of interruption.

Bridge financing requires a costed protected package, identified funding, end-point and handover. Humanitarian, development, pooled or domestic/Benadir resources are conditional: donor commitments may restrict reprogramming, which depends on contractual flexibility [1]. Without sufficient funding, prioritise and sequence rather than promise unfunded services.

Match support to bottlenecks: direct subsidies for capable public/non-profit providers; auditable strategic purchasing when public capacity is insufficient; medicine or transport assistance where supply exists and costs obstruct access. Household support may connect to shock-responsive protection, but Baxnaano eligibility, reach and suitability for displaced people require assessment [13,18].

Private purchasing requires credentialing, quality checks, published benefits/tariffs, no balance billing, auditable payments, sanctions and independent complaints. A legally competent purchaser or authorised independent verifier identified through governance mapping should verify providers and investigate billing; no institutional mandate is presumed. Contracts must protect referral continuity and payment timeliness: delayed reimbursement risks informal charges, rationing or refusal [11,12,17].

## Mogadishu political economy, equity and community accountability

Settlement managers may mediate aid and land in Mogadishu [4] but must not control eligibility, beneficiary lists, complaints or verification. Community-health-worker, women’s-group and disability-organisation outreach, rotating contacts and independent grievance channels should protect against coercion, exclusion and diversion.

The framework targets internally displaced people, including mobile, newly displaced, unregistered and disability/chronic-care populations outside formal rosters [3,4,13,18]. Eligibility should be need-based and reassessed. Host-community or geographic expansion requires separate eligibility and financing decisions. Communities should influence protected services, costs, complaints and provider accountability [19].

## Minimum viable monitoring and implementation responsibilities

Monitoring should measure both hardship and exclusion while remaining feasible (Table 1). Facility/contract systems should report service continuity, prescription fill, priority referral completion and provider-payment timeliness. Quarterly sentinel household surveys should include distress financing and, where feasible, catastrophic/impoverishing expenditure [6,7]. Financial exclusion should be reported separately as delayed, incomplete and completely forgone care; any combined summary should be secondary because these outcomes represent different degrees of non-receipt [8]. Treatment interruption should have separate population- and facility-based indicators. For affected episodes, record whether the barrier reflects inability to pay, expected unaffordability or another cost-sensitive reason, distinguishing consultation/registration, medicine, diagnostics, transport/referral, informal payments, anticipated costs and lost wages/opportunity costs. Use a three-month recall for unauthorised charges, hardship and cost-related access outcomes. Table 1 specifies the numerators, denominators, data sources and frequencies so household-survey and facility indicators are not merged across populations.

**Table 1.** Proposed financial-protection transition matrix and minimum viable monitoring set for humanitarian health-service contraction in Mogadishu.

Transition risk	Selection trigger and minimum proposed action	Evidence/rationale	Minimum viable monitoring indicator, source and frequency
Service contraction and exposure	Trigger: planned or observed reduction in financing, service days, catchment coverage, commodities, mobile teams or outreach. For planned reductions, conduct a pre-exit continuity assessment. For abrupt withdrawal, rapidly map lost services and affected populations, prioritise time-sensitive gaps and temporarily restore critical functions where feasible. Protect a costed essential package using explicit clinical and equity criteria.	Somalia operational and policy evidence [1–3,17]; authors’ proposed sequencing and exposure definition.	Exposure (% vs comparable baseline): funding fall = (baseline − current)/baseline; service-day loss = (baseline scheduled − delivered)/baseline scheduled days; tracer-supply loss = (baseline − supplied)/baseline units (per item); outreach-day loss = (baseline planned − delivered)/baseline planned days. Count team closures separately. Service continuity = delivered/scheduled days. Sources: finance, facility, supply and outreach records; monthly and pre/post cuts.
Medicine or diagnostic gaps	Trigger: recurrent stock-outs, reduced commodity support or incomplete dispensing. Finance tracer commodities; use regulated purchasing or vouchers only when supply exists and the cost barrier is verified.	Somalia access and policy evidence [3,5]; fragile-setting financing literature [11,12] and humanitarian user-fee guidance [14]; authors’ proposed application.	Prescription fill = tracer prescriptions fully dispensed/tracer prescriptions issued. Source: pharmacy/dispensing log. Monthly.
Transport or referral failure	Trigger: falling priority referral completion or repeated transport-cost complaints. Protect referral links and target transport support to verified barriers.	Somalia displaced-population evidence [4,5]; authors’ proposed trigger.	Priority referral completion = completed priority referrals/initiated priority referrals. Source: referral register plus receiving-facility confirmation. Monthly.
New or unauthorised charges	Trigger: reports from users of fees outside the protected package or contracted tariff. Fund exemptions or replacement revenue; publish tariffs; prohibit balance billing; investigate violations.	Humanitarian user-fee guidance [14]; Somali financing constraints [3].	Unauthorised charge rate = interviewed users reporting a non-authorised charge in the preceding 3 months/interviewed users of protected services during the same preceding 3-month period. Source: community or exit sample and grievance log. Quarterly.
Household financial hardship	Trigger: rising household financial strain attributable to obtaining care. Review fees, medicines, diagnostics, transport and household support; add fuller expenditure measurement where feasible.	WHO financial-protection guidance [6] and financial-risk literature [7]; authors’ proposed minimum indicator.	Distress financing = households reporting borrowing, asset sales or use of essential savings for health spending in the preceding 3 months/households reporting health spending in that period. Source: household sentinel survey. Quarterly. Add catastrophic or impoverishing expenditure if a fuller expenditure module is feasible.
Cost-related financial exclusion	Trigger: rising cost-related delay, incomplete care or complete non-use of care. Review the specific bottleneck. Record whether the reason is an inability to pay, expected unaffordability or another cost-sensitive reason, and disaggregate consultation/registration, medicines, diagnostics, transport/referral, informal payments, anticipated total costs and lost wages/opportunity costs.	WHO measurement guidance [6,8] and supporting financial-protection and Somalia evidence [7,9,10].	Report separately: delayed care = need episodes with cost-related delay/all need episodes; incomplete care = cost-related partly met episodes/all need episodes; completely forgone care = cost-related unmet episodes/all need episodes. A combined financial-exclusion summary may be secondary. Source: household sentinel survey; preceding 3-month recall period. Quarterly.
Treatment interruption among priority patients	Trigger: interruption of required ongoing treatment because support for services, medicines, referrals or costs is lost. Restore continuity or targeted support.	WHO measurement framework [8] and authors’ proposed application.	Population interruption = surveyed people needing ongoing treatment who report interruption in prior 3 months/surveyed people needing ongoing treatment in that period. Facility interruption = registered priority patients with documented interruption during quarter/registered priority patients expected to continue treatment that quarter. Report separately. Sources: household sentinel survey and clinic register. Quarterly.
Unfinanced exit or contracted-provider payment failure	Trigger: planned reduction without financed replacement, abrupt withdrawal without continuity arrangements, or overdue contracted-provider reimbursement. Planned exit: require costed handover and financing-source identification. Abrupt exit: rapid gap assessment, emergency prioritisation and feasible temporary restoration. Payment delay: escalate arrears before informal charging, rationing or refusal emerges.	Somalia funding and health-financing evidence [1,2,17]; fragile-setting financing literature [11,12]; and authors’ proposed transition safeguards.	Financing coverage = funding committed/cost of protected transition package. Provider payment timeliness = payments made by the contractual due date/payments due. Sources: financing and contract records. Quarterly and before major reductions; payment timeliness monthly.

Note: Transition risks are evidence-informed hypotheses and the actions are proposed policy options, not observed effects of funding contraction in Mogadishu. The framework is IDP-targeted. Household sentinel surveys should retain a stable core of locations for comparability and report rotating outreach locations separately to reach mobile, newly displaced, unregistered and other people who might otherwise be missed. Alert thresholds should be prespecified locally against baseline values, agreed tolerances, clinical importance and persistence across reporting periods. Overlapping care-episode outcome rates must be reported separately, not summed. Indicators should be piloted before scale-up.

For comparability retain a stable sentinel-site core, reporting rotating outreach separately. Sample catchments with differing exposure; recruit through community-health-worker contacts, water points, markets, transport nodes and mobile services to include those turned away, unable to travel, newly displaced, unregistered or excluded from lists. Without probability sampling, results describe changing barriers, not citywide prevalence.

Governance assignments are proposed functions, not claims of existing statutory mandates. Before implementation, mapping should establish legal/policy authority for benefits, contracting, fund control, data access, claims verification, billing investigations and sanctions, and identify functions needing delegation or new contracts. Subject to that mapping, functions could include federal health authorities setting protected-package and purchasing standards; Benadir authorities coordinating provider mapping, contracting and monitoring; funders and Health Cluster partners providing notice of reductions and financing visibility; social-protection authorities supporting feasible Baxnaano linkage; providers reporting service/claims data; and independent verification mechanisms and grievance channels reviewing performance. Contracts should specify payment schedules, documentation, fraud controls, escalation procedures and responsibility for financing failure.

Prespecify local action thresholds using baselines, agreed tolerances, clinical significance and deterioration sustained across consecutive reporting periods; severe closures or safety-critical interruptions merit immediate action. Collect minimal consent-based data, restrict settlement identifiers and aggregate public reporting [20].

## Scope, risks and limitations

This purposive policy analysis is non-systematic and non-empirical; it may omit unpublished, local or non-English evidence and lacks risk-of-bias assessment. National/multi-location data cannot establish Mogadishu household effects of the 2025–2026 contraction; concurrent shocks complicate attribution.

The framework remains untested, uncosted and not co-designed with displaced people, providers, authorities or funders; its acceptability, feasibility, costs, cost-effectiveness and implementation effectiveness are unknown. Financing remains unsecured, mandates unmapped and thresholds unvalidated. Purchasing safeguards cannot eliminate arrears-related charging, rationing or refusal. Local adaptation and prospective evaluation are essential; transfer beyond Mogadishu depends on comparable financing, displacement, access costs and regulatory capacity.

## Conclusion

Unfunded reductions in subsidised services may shift costs to households, but causal effects of the 2025–2026 contraction in Mogadishu remain unproven. The proposed framework jointly measures financial hardship and distinct delayed, incomplete and completely forgone care. Planned withdrawal requires funded continuity; abrupt withdrawal demands rapid gap assessment and emergency prioritisation. Transparent essential-service selection, accountable purchasing, protection against gatekeeping and prespecified monitoring alerts require prospective, community-engaged evaluation.

## Data Availability

No new datasets were generated or analysed; supporting documents are cited.
